# Generation of *GHR*-modified pigs as Laron syndrome models via a dual-sgRNAs/Cas9 system and somatic cell nuclear transfer

**DOI:** 10.1186/s12967-018-1409-7

**Published:** 2018-02-27

**Authors:** Honghao Yu, Weihu Long, Xuezeng Zhang, Kaixiang Xu, Jianxiong Guo, Heng Zhao, Honghui Li, Yubo Qing, Weirong Pan, Baoyu Jia, Hong-Ye Zhao, Xingxu Huang, Hong-Jiang Wei

**Affiliations:** 1 School of Life Science and Technology, ShanghaiTech University, 100 Haike Rd., Pudong New Area, Shanghai, 201210 China; 2 State Key Laboratory for Conservation and Utilization of Bio-Resources in Yunnan, Yunnan Agricultural University, Kunming, 650201 China; 3 College of Animal Science and Technology, Yunnan Agricultural University, Kunming, 650201 China; 4 College of Veterinary Medicine, Yunnan Agricultural University, Kunming, 650201 China; 5 College of Biotechnology, Guilin Medical University, Guilin, 541100 China

**Keywords:** *GHR* knockout, Dual-sgRNAs/Cas9, Laron syndrome, SCNT, *Diannan* miniature pig

## Abstract

**Background:**

Laron syndrome is an autosomal disease resulting from mutations in the growth hormone receptor (*GHR*) gene. The only therapeutic treatment for Laron syndrome is recombinant insulin-like growth factor I (IGF-I), which has been shown to have various side effects. The improved Laron syndrome models are important for better understanding the pathogenesis of the disease and developing corresponding therapeutics. Pigs have become attractive biomedical models for human condition due to similarities in anatomy, physiology, and metabolism relative to humans, which could serve as an appropriate model for Laron syndrome.

**Methods:**

To further improve the *GHR* knockout (GHRKO) efficiency and explore the feasibility of precise DNA deletion at targeted sites, the dual-sgRNAs/Cas9 system was designed to target *GHR* exon 3 in pig fetal fibroblasts (PFFs). The vectors encoding sgRNAs and Cas9 were co-transfected into PFFs by electroporation and GHRKO cell lines were established by single cell cloning culture. Two biallelic knockout cell lines were selected as the donor cell line for somatic cell nuclear transfer for the generation of GHRKO pigs. The genotype of colonies, cloned fetuses and piglets were identified by T7 endonuclease I (T7ENI) assay and sequencing. The GHR expression in the fibroblasts and piglets was analyzed by confocal microscopy, quantitative polymerase chain reaction (q-PCR), western blotting (WB) and immunohistochemical (IHC) staining. The phenotype of GHRKO pigs was recapitulated through level detection of IGF-I and glucose, and measurement of body weight and body size. GHRKO F1 generation were generated by crossing with wild-type pigs, and their genotype was detected by T7ENI assay and sequencing. GHRKO F2 generation was obtained via self-cross of GHRKO F1 pigs. Their genotypes of GHRKO F2 generation was also detected by Sanger sequencing.

**Results:**

In total, 19 of 20 single-cell colonies exhibited biallelic modified *GHR* (95%), and the efficiency of DNA deletion mediated by dual-sgRNAs/Cas9 was as high as 90% in 40 *GHR* alleles of 20 single-cell colonies. Two types of *GHR* allelic single-cell colonies (*GHR*^−*47/*−*1*^, *GHR*^−*47/*−*46*^) were selected as donor cells for the generation of GHRKO pigs. The reconstructed embryos were transferred into 15 recipient gilts, resulting in 15 GHRKO newborn piglets and 2 fetuses. The GHRKO pigs exhibited slow growth rates and small body sizes. From birth to 13 months old, the average body weight of wild-type pigs varied from 0.6 to 89.5 kg, but that of GHRKO pigs varied from only 0.9 to 37.0 kg. Biochemically, the knockout pigs exhibited decreased serum levels of IGF-I and glucose. Furthermore, the GHRKO pigs had normal reproduction ability, as eighteen GHRKO F1 piglets were obtained via mating a GHRKO pig with wild-type pigs and five GHRKO F2 piglets were obtained by self-cross of F1 generation, indicating that modified *GHR* alleles can pass to the next generation via germline transmission.

**Conclusion:**

The dual-sgRNAs/Cas9 is a reliable system for DNA deletion and that GHRKO pigs conform to typical phenotypes of those observed in Laron patients, suggesting that these pigs could serve as an appropriate model for Laron syndrome.

**Electronic supplementary material:**

The online version of this article (10.1186/s12967-018-1409-7) contains supplementary material, which is available to authorized users.

## Background

Patients of Laron syndrome, an autosomal disease showing growth hormone (GH) resistance, are characterized as having very short stature, small midface, frontal bossing, small genitalia and truncal obesity [1]. Currently, the pathogenesis of Laron syndrome has been confirmed as mutations of the GH receptor (*GHR*). Mutations in the extracellular domain of GHR protein result in an inability to bind GH ligands and are found in most Laron patients, and other mutations result in the inability to transfer signals [2, 3]. In addition, Laron patients carrying mutations in the *GHR* gene exhibit severe congenital insulin-like growth factor I (IGF-I) deficiency [4]. To date, the only therapeutic treatment for Laron syndrome is recombinant IGF-I, which has been shown to have various side effects, including hypoglycemia, thymic hypertrophy, snoring and hypoacusis [5].

To investigate mechanisms of Laron syndrome and develop therapeutic options, several animal models have been proposed, including dwarf chickens and Laron mice [6–8]. While dwarf chicken models present a significant reduction in body weight and some signs of GH resistance, they are not the ideal models of Laron syndrome due to the large taxonomic difference between birds and mammals and their discrepant GH axes [9]. The Laron mice mimic in many respects the human syndrome, including severe postnatal growth retardation, proportionate dwarfism, absence of the GHR binding protein, greatly decreased serum IGF-I and elevated serum GH concentrations [8]. However, the Laron syndrome belongs to metabolic disease, of which hormone regulatory pathways are fundamentally different between human and rodents [10]. In addition, Kopchick and Laron hoped that Laron animal model would enable the elucidation of many aspects of longstanding IGF-I derivation on a variety of organs and tissues [11], thus, Laron mice is not suitable for such investigation due to their small body size and short life-span. The pig is an ideal animal model for human diseases because their physiological and anatomical similarities to humans. Recently, a spectrum of pig models for metabolic, cardiovascular, infectious diseases, xenotransplantation and neurological disorders have been generated [12], suggesting that the modified pig models might more precisely recapitulate the phenotype of Laron syndrome or bridge the gap between classical rodent models and humans in such disease.

The CRISPR/Cas9 system has been demonstrated to be a highly efficient genome editing tool for generating gene-modified animal models [13–17]. Our previous success in generating genetically modified pigs using the CRISPR/Cas9 system [15, 17, 18] demonstrates the great potential of CRISPR/Cas9 to genetically modify pigs. Random short deletions or insertions (indels) occur because of non-homologous end joining induced by DNA double-stranded breaks introduced by CRISPR/Cas9, and indels occurring in coding regions can cause null mutations of targeted genes. A few reports have described gene replacement [19] and deletions ranging from 1.3 kilobases (kb) to more than one megabase (1 Mb) in length [20–22] using designed dual sgRNAs, and these reports inspired the creation of precise DNA deletion mediated by a dual-sgRNAs/Cas9 system.

Here, we successfully created DNA deletion in targeting sites, resulting in *GHR* knockout (GHRKO) pigs via a dual-sgRNAs/Cas9 system mediated by DNA deletion combined with somatic cell nuclear transfer (SCNT) and determined whether pigs lacking *GHR* could function as animal models by recapitulating the phenotypes of human Laron syndrome.

## Methods

### Chemicals

All chemicals were purchased from Sigma Chemical Co. (St. Louis, MO, USA) unless otherwise stated.

### Animals

*Diannan* miniature pigs are a breed exclusively native to the Yunnan Province of China and have suitable full-grown body weights, which makes this strain an ideal animal for generating human disease models [23, 24]. The miniature pigs used in our study were regularly maintained at the Animal Center of Yunnan Agricultural University. All experiments involving pigs were approved by the Institutional Animal Care and Use Committee of Yunnan Agricultural University (permission code: YAUACUC01; publication date: 10 July 2013).

### Design of dual sgRNAs targeting *GHR* and construction of the plasmid vectors

Dual sgRNAs targeting *GHR* exon 3 were designed as described previously [25] to disrupt the function of *GHR*. Meanwhile, one sgRNA targeting *lacZ* (sgRNAc) was also designed for control. Three pairs of complementary DNA oligos for generating sgRNAs, listed in Additional file 1: Table S1, were annealed. Subsequently, the double-stranded DNA coding regions of the three sgRNAs were subcloned into the pGL3-U6-sgRNA vector (Addgene no: 51133) as previously described [26]. The constructed pGL3-U6-GHR-sgRNA1, pGL3-U6-GHR-sgRNA2 and PGL3-U6-Con-sgRNAc plasmid vectors were confirmed by sequencing. We then prepared the high concentration and endotoxin-free pGL3-U6-GHR-sgRNA1, pGL3-U6-GHR-sgRNA2, pGL3-U6-Con-sgRNAc and pST1374-NLS-flag-linker-Cas9 (Addgene no: 44758) plasmids for cell transfection.

### Cell culture, transfection and selection

Pig fetal fibroblasts (PFFs) were isolated from 35-day-old *Diannan* miniature pig fetuses, cultured in T25 flasks for 48 h, and then frozen in Dulbecco’s Modified Eagle’s Medium (DMEM, Thermo Fisher Scientific, China) supplemented with 10% dimethyl sulfoxide and 20% FBS for later use. One day before PFF transfection, PFFs were thawed and cultured in DMEM supplemented with 20% FBS. Approximately 4 × 10^6^ PFFs in 200 μl of electroporation buffer containing pGL3-U6-GHR-sgRNA1 (5 μg), pGL3-U6-GHR-sgRNA2 (5 μg) and pST1374-NLS-flag-linker-Cas9 (5 μg) were electroporated at 250 V for 20 ms with a Gene Pulser Xcell electroporator (Bio-Rad, California, USA).

After electroporation, the cells were placed into T25 flasks for 24 h in DMEM supplemented with 20% FBS. Then, 3 μg/ml puromycin and 5 μg/ml blasticidin S were added to the medium for 24–48 h to select successfully transfected cells. The surviving cells were then digested, and approximately 80 cells were seeded into 10-cm culture dishes for 8–12 days. Subsequently, the cell colonies were seeded individually into 48-well plates to isolate single colonies. Finally, single colonies harvested from 48-well plates were subsequently passaged to 24-well plates and 12-well plates. Single cell-derived colonies harvested from 12-well plates after 3 d of culture were frozen for later use. A fraction of colonized cells were genotyped by PCR and subjected to a T7 endonuclease I (T7ENI) cleavage assay and sequencing for the identification of biallelic *GHR* knockouts. The identified biallelic *GHR* knockout cell colonies were selected as donor cells for nuclear transfer.

For confirmation of DNA fragment deletion induced by dual sgRNAs targeting GHR, the pig iliac endothelium (PIEC) cell line (a gift from ShanghaiTech University) was culture above described conditions and transfected with the plasmids encoding sgRNAs and Cas9 protein. One group containing pGL3-U6-GHR-sgRNA1 (10 μg), pGL3-U6-Con-sgRNAc (10 μg) and pST1374-NLS-flag-linker-Cas9 (20 μg), and another group including pGL3-U6-GHR-sgRNA2 (10 μg), pGL3-U6-Con-sgRNAc (10 μg) and pST1374-NLS-flag-linker-Cas9 (20 μg) were electroporated, respectively, at 140 V for 1000 ms with an X-Porator EBXP-H1 electroporator (Etta Biotech, Suzhou, China). Subsequently, 1.5 μg/ml puromycin were added into medium for 48 h for selection of transfected cells. Then the survived cells were collected for T7ENI cleavage assay and TA cloning sequencing.

### Somatic cell nuclear transfer (SCNT) and generation of GHRKO piglets

Pig ovaries were obtained from Hongteng slaughterhouse (Chenggong Ruide Food Co., Ltd, Kunming, Yunnan Province, China). Cumulus-oocyte complexes (COCs) with at least three layers of compacted cumulus cells aspirated from follicles 3–6 mm in diameter were selected for in vitro maturation (IVM) at 38.5 °C in a saturated humidified atmosphere of 5% CO_2_ (APC-30D, ASTEC, Japan) for 42–44 h.

After IVM, COCs with expanded cumulus cells were treated with 0.1% (w/v) hyaluronidase, and the cumulus cells were removed by gentle pipetting. COC-free oocytes were enucleated by aspirating the first polar body and adjacent cytoplasm using a micromanipulator system. Biallelic GHRKO cells were injected into the perivitelline space of enucleated oocytes and then fused with recipient cytoplasts in fusion medium using a single direct current pulse of 200 V/mm for 20 μs with an embryonic cell fusion system (LF 201, Nepa Gene Co. Ltd., Tokyo, Japan). The reconstructed embryos were cultured for 2 h in porcine zygote medium-3 (PZM-3) and then activated with a single pulse of 150 V/mm for 100 μs in an activation medium [27]. The reconstructed embryos were equilibrated in PZM-3 supplemented with 5 μg/ml cytochalasin B for 2 h at 38.5 °C in a humidified atmosphere of 5% CO_2_, 5% O_2_ and 90% N_2_ (APM-30D, ASTEC, Japan). Then, the embryos were washed three times with PZM-3 medium and cultured in PZM-3 medium under the same conditions described above.

Crossbred prepubertal gilts (large white/landrace duroc) weighing 100–120 kg were used as surrogates for the reconstructed embryos. Reconstructed embryos cultured for 2–30 h after activation were surgically transferred to the oviducts of the surrogates. Pregnancy was determined by abdominal ultrasound examination 23 days after the embryo transfer. Approximately 114 days later, piglets were delivered by natural birth or Caesarean section.

### Genotyping

For analysis of the *GHR* genotype, cells grown from established single-cell colonies or PIEC cell were lysed in 10 μl of NP-40 solution for 15 min at 65 °C and 10 min at 95 °C. Cell lysates were used as templates for PCR amplification of the targeted region with specific primers (Additional file 1: Table S2). PCR products were purified using a cleanup kit (AP-PCR-50, Axygen, New York, USA), and the purified PCR product mixtures (100 ng of the wild-type PCR product mixed with 100 ng of the *GHR*-targeted PCR product) were denatured and reannealed in NEBuffer 2 (NEB, Massachusetts, USA) using a thermocycler. Denatured and reannealed products were digested with T7ENI (M0302 L, NEB, Massachusetts, USA) for 30 min at 37 °C and detected by electrophoresis in a 2% agarose gel. PCR products in which mutations were determined by the T7ENI cleavage assay were sub-cloned into the pMD19 T vector (Takara, Dalian, China) for sequencing. The M13F primer (-47, 5′-CGCCAGGGTTTTCCCAGTCACGAC-3′) was used for sequencing.

For analysis of the *GHR* genotype in the obtained piglets, ear tissues collected from piglets were digested in lysis buffer (0.4 M NaCl, 2 µM EDTA, 1% SDS, 10 µM Tris–HCl, and 100 µg/ml proteinase K) overnight. Genomic DNA of the sample was extracted from the lysates with phenol–chloroform and recovered by ethanol precipitation. The T7ENI cleavage assay and Sanger sequencing were performed as described above.

### Quantitative RT-PCR analysis

Total RNAs for quantitative polymerase chain reaction (q-PCR) analysis were extracted from the hearts, livers, kidneys and muscles of four GHRKO pigs and three WT pigs. cDNA was synthesized based on 1 μg of RNA template with an available kit (TaKaRa Biotech). q-PCR was set up in 20 μl reaction mixtures comprising 10 μl of 2× SYBR (TaKaRa Biotech), 1 μl of cDNA, 1 μl of forward primer, 1 μl of reverse primer, and 7 μl of ddH_2_O. The reaction program was as follows: 95 °C for 3 min, followed by 40 cycles of 95 °C for 10 s and 55 °C for 30 s. Porcine *GAPDH* was used for normalization. Target genes expession was quantified using the comparative cycle threshold methold. The primers are listed in Additional file 1: Table S3.

### Fluorescence microscopy

Fibroblasts isolated form GHRKO piglets and control fibroblasts isolated from wild-type piglets were cultured on glass coverslips for 24 h, fixed with 4% paraformaldehyde for 10 min, and washed with PBS. The fixed cells were immersed in 0.2% Triton X-100 for 10 min and washed with PBS. The cells were then blocked with 1% bovine serum albumin in PBS for 1 h at room temperature and then incubated overnight in a 6-well plate at 4 °C with anti-GHR (1:100 v/v, ab202964, Abcam, Cambridge, England) in blocking buffer. Subsequently, the cells were washed with PBS, incubated with anti-rabbit secondary antibodies (1:400 v/v, Jackson ImmunoResearch, West Grove, USA) for 30 min at room temperature, and washed with PBS once again. Nuclei were counterstained with 1 μg/ml 6-diamidino-2-phenylindole (DAPI). The slides were covered with mounting medium and observed under a laser scanning confocal microscope (OLYMPUS FV1000, Tokyo, Japan).

### Immunohistochemical analysis of tissue sections

GHRKO piglets and wild-type piglets were euthanized by CO_2_ inhalation, and their hearts, livers, kidneys, spleens, testes and muscles were excised. These tissues were fixed using 4% paraformaldehyde at room temperature. A graded ethanol series was used to dehydrate the tissues, which were then embedded in paraffin. Paraffin-embedded tissues were sectioned at a thickness of 5–8 μm. The sections were antigen-repaired with heat and blocked with endogenous peroxidase. The sections were then blocked by 5% bovine serum albumin in PBS for 15 min at 37 °C and incubated overnight in a humid chamber at 4 °C with anti-GHR (1:400 v/v, ab202964, Abcam, Cambridge, England) in blocking buffer. The next day, the sections were washed three times with PBS and subsequently incubated with a horseradish peroxidase (HRP)-conjugated secondary antibody (ZSGB-BIO, Ltd., China). The sections were visualized with 3,3′-enzidine tetrahydrochloride (ZSGB-BIO, Ltd., China), and the nuclei were counterstained with hematoxylin. Finally, the slides were examined via microscopy (Leica, DM2000, Germany).

### Protein extraction and immunoblotting

Protein extraction and immunoblotting were performed as described in our previous study [28]. Hearts and kidneys from GHRKO piglets and wild-type piglets were used to evaluate GHR protein levels using western blot analysis. In brief, tissues were lysed using RIPA lysis buffer (Bestbio, China) with protease inhibitors at 4 °C. After lysis, supernatants were collected by centrifugation at 12,000× rpm for 15 min at 4 °C. Equal amounts of protein (60 μg) and a protein weight marker were separated by SDS-PAGE. The proteins were then transferred to polyvinylidene difluoride (PVDF) membranes and incubated with primary antibodies against GHR (1:1000 v/v, ab202964, Abcam, Cambridge, England) and β-actin (1:5000 v/v, Sigma-Aldrich;) at 4 °C overnight. After incubation, the membranes were washed and reacted with anti-mouse or anti-rabbit secondary antibodies (R&D, USA). The membranes were then incubated with enhanced chemiluminescence (ECL) (Easysee Western Blot Kit, China) and visualized with an imaging system (Bio-Rad).

### Measurement of growth parameters

We documented the parameters of GHRKO piglets and wild-type piglets from newborn to 13 months old. The growth parameters analyzed included body weight, body length, withers height, chest depth, chest circumference, abdominal circumference, hip length, hip width, cannon circumference, chest width, head length, forehead width, tail length and tail circumference.

### Enzyme-linked immunosorbent assay (ELISA)

Blood samples were collected from GHRKO pigs and wild-type pigs after they were fasted overnight, and sera were collected by centrifugation at 4 °C for 5 min at 3000× rpm and frozen at − 80 °C for future assays. The levels of IGF-I in both GHRKO pigs and control pigs were determined using the enzyme-linked immunosorbent assay (ELISA) kit for porcine IGF-I (USCN, China) according to the manufacturer’s instructions. To assay the amounts of IGF-I in serum, sera were diluted at 1: 40. Briefly, 100 μl of the diluted serum samples were added to pre-coated wells. After incubation for 2 h at 37 °C, 100 μl of detection antibody was added for 1 h at 37 °C, followed by washing. Then, 90 μl of HRP solution was added to the mixtures, and the colorimetric reaction was subsequently induced with 50 μl of the 3,3′,5,5′-tetramethylbenzidine (TMB) substrate. The absorbance of the products was measured at 450 nm by a microplate reader (Bio-Rad). In addition, fasting blood glucose levels were measured after fasting overnight using Accu-CHEK performa test strips (Roche, Germany).

### Germline transmission of *GHR*-modified alleles

Germline transmission of modified *GHR* alleles to the next generation is essential for expanding the population of GHRKO pigs to further investigate the pathomechanism and therapeutic options. A male GHRKO pig (P2) cloned from the single-cell clone C3 mated with nature estrus female wild-type pigs, and their offspring were delivered by natural birth. The genotypes of the GHRKO F1 generation were detected as described above. To breed pigs harboring allelic mutation in *GHR* loci by germline transmission and certify the modified *GHR* stable multi-generational transmission, GHRKO F2 generation was obtained via self-cross of GHRKO F1 piglets. The genotypes of GHRKO F2 generation were also detected by Sanger sequencing.

### Statistical analysis

All data were expressed as the mean ± standard deviation (SD). Independent sample *t*-tests were performed using the SPSS 22.0 software package (IBM 230 Corp., Armonk, NY, USA). Statistical significance was defined as *P < 0.05 and **P < 0.01.

## Results

### Designed dual-sgRNAs/Cas9 system was efficiently employed to disrupt *GHR* in PFFs

**Fig. 1:**
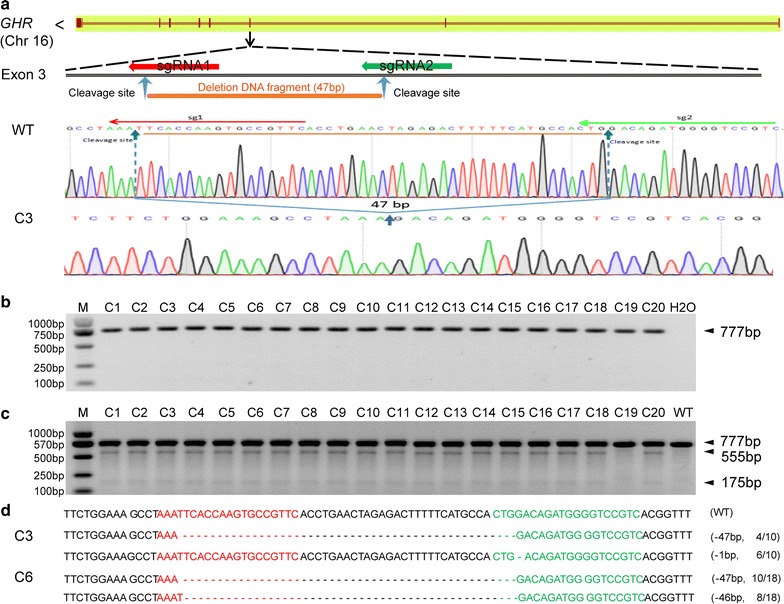
Establishment of *GHR*-modified single-cell colonies. **a** Schematic diagram of DNA deletion mediated by the dual-sgRNAs/Cas9 system. The brown line indicates the *GHR* gene structure from the Ensmbl database. “<” indicates the direction of transcription. The red and green arrows indicate the targeting sites of the designed sgRNAs. The blue arrows indicate cleavage sites mediated by the sgRNAs/Cas9 system. The orange segment and line indicate the deleted 47-bp DNA fragment mediated by the dual-sgRNAs/Cas9 system. WT: wild-type allele. C3: single-cell colony #3. **b** PCR products harboring the targeted *GHR* region amplified from pig single-cell colonies (M, DNA maker DL2000; C, colony). **c** Detection of sgRNAs/Cas9-mediated on-target *GHR* cleavage by the T7ENI cleavage assay. The band size of wild-type PCR product is 777 bp, and the sizes of T7ENI cut bands are about 555 and 175 bp. The mismatched about 47 bp band was not detected. **d** Sequence of modified GHR in donor cells for SCNT. The red font indicates the sgRNA1 targeting site, and the green font indicates the sgRNA2 targeting site. C6: single-cell colony #6. “–”: deletion; N/N: positive sequencing out of total sequencing

**Fig. 2:**
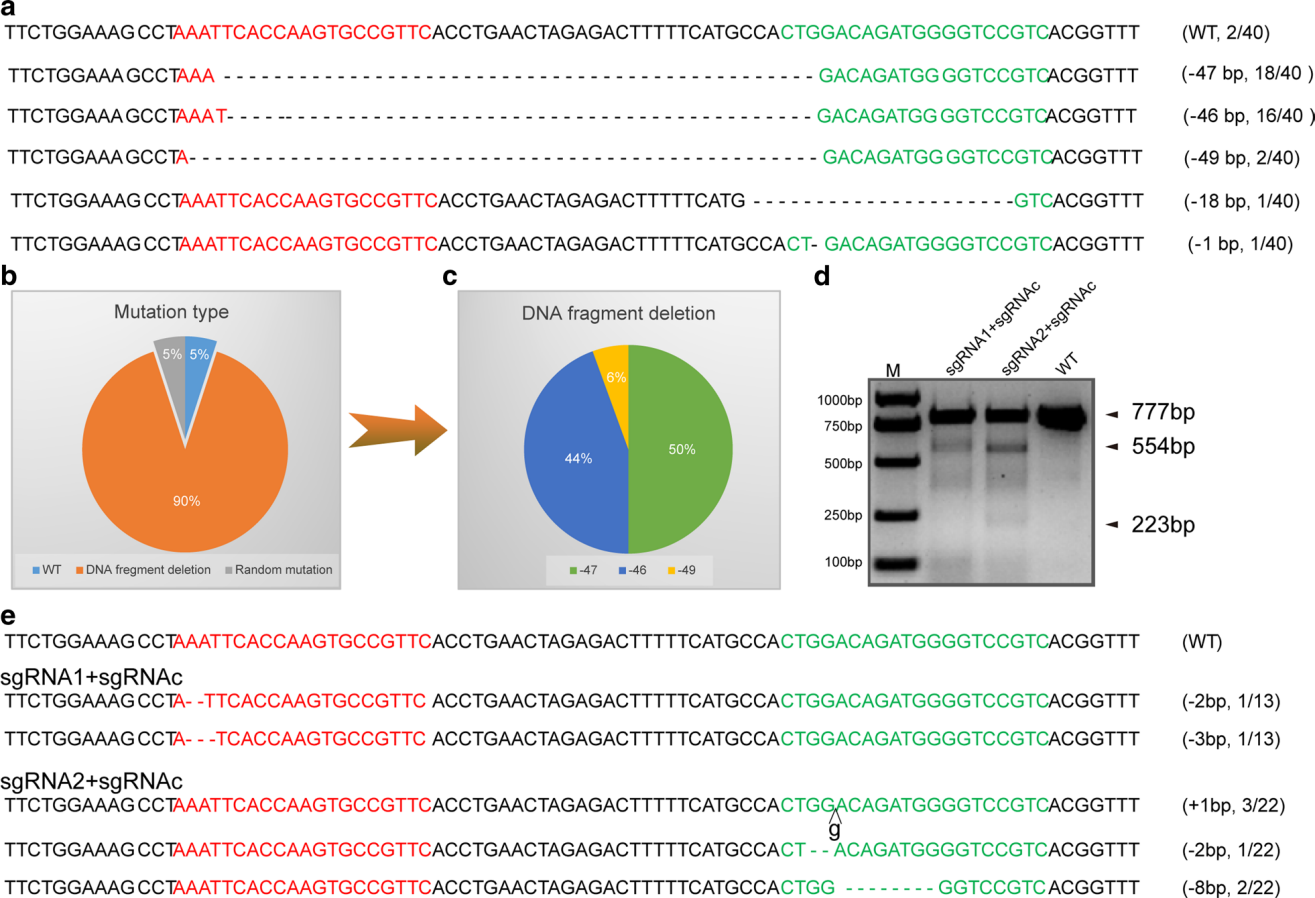
Highly efficient DNA deletion mediated by dual-sgRNAs/Cas9. **a** Detected modified *GHR* sequences of 40 alleles in 20 single-cell colonies. The five types of mutations detected include − 47, − 46, − 49, − 18 and − 1 bp. WT: wild-type sequence. “–”: deletion. N/40: Modified *GHR* alleles detected out of 40 total alleles. **b** Percentage of *GHR* mutation types detected in 40 alleles. **c** Percentage of DNA fragment deletion. **d** Detection of sgRNA1 and sgRNA2 programmed Cas9 targeting effect by T7ENI cleavage assay. **e** The mutant genotype of *GHR* mediated by sgRNA1/Cas9 or sgRNA2/Cas9 system. The red font indicates the sgRNA1 targeting site, and the green font indicates the sgRNA2 targeting site

To improve the biallelic *GHR* modification efficiency and explore the feasibility of precise DNA deletion at targeted sites, we designed dual sgRNAs to target exon 3 of *GHR*; the distance of two cleavage sites mediated by two dual-sgRNAs/Cas9 is was 47 nucleotides (Fig. 1a). We co-transfected the dual sgRNAs and Cas9 plasmids into PFFs, calculated the efficiency of *GHR* modification and analyzed the *GHR* genotype by the T7ENI cleavage assay, TA cloning and Sanger sequencing. A total of 20 single-cell colonies were obtained after puromycin and blasticidin S selection. All modifications of *GHR* alleles in 19 single-cell colonies were biallelic, indicating an efficiency of *GHR* biallelic modification as high as 95% (Fig. 1b–d). We detected a total of 5 types of *GHR* modification by TA cloning and Sanger sequencing in 40 *GHR*-modified alleles of 20 single-cell colonies, including − 46 bp deletion, − 47 bp deletion, − 49 bp deletion, − 18 bp deletion and − 1 bp deletion (Fig. 2a and Additional file 1: Table S4). DNA deletions (46, 47, and 49 bp) were successfully achieved using the dual-sgRNAs/Cas9 system with a 90% efficiency (Fig. 2b). The CRISPR/Cas9 gene editing system cleaves double-stranded DNA at the third nucleotide neighboring the 5′ region of the protospacer adjacent motif (PAM). In our study, a 47-bp nucleotide sequence harboring the two cleavage sites was mediated by the designed dual sgRNAs. We detected a 47-bp deletion precisely mediated by the dual-sgRNAs/Cas9 system, with an efficiency as high as 50% (Fig. 2c). Further analysis of the targeted site sequence showed that the 18- and 1-bp deletion modifications were mediated by only the second guide RNA/Cas9 combination (sgRNA2/Cas9) (Fig. 1a and Additional file 1: Table S4).

We speculated that synergistic effect of dual-sgRNAs/Cas9 system induce DNA fragment deletion and improve the targeting efficiency. Therefore, one sgRNAc targeting *lacZ* (sgRNAc) was designed used as control for co-transfection with sgRNA1 or sgRNA2 and Cas9 in PIEC cell line. The result showed that both sgRNA1 and sgRNA2 have the ability of targeting cleavage (Fig. 2d). However, the targeting efficiency was dramatically decreased when sgRNA1 or sgRNA2 programmed Cas9 alone. 15.4% (2/13) and 27.3% (6/22) mutation were detected by further analysis of genotype (Fig. 2e), the targeting efficiency is reduced approximately 3.5-fold compared with that of sgRNA1 combined with sgRNA2. Overall, the above results strongly suggest that the dual-sgRNAs/Cas9 is a reliable system for DNA deletion.

### Generation of GHRKO pigs by SCNT

**Fig. 3:**
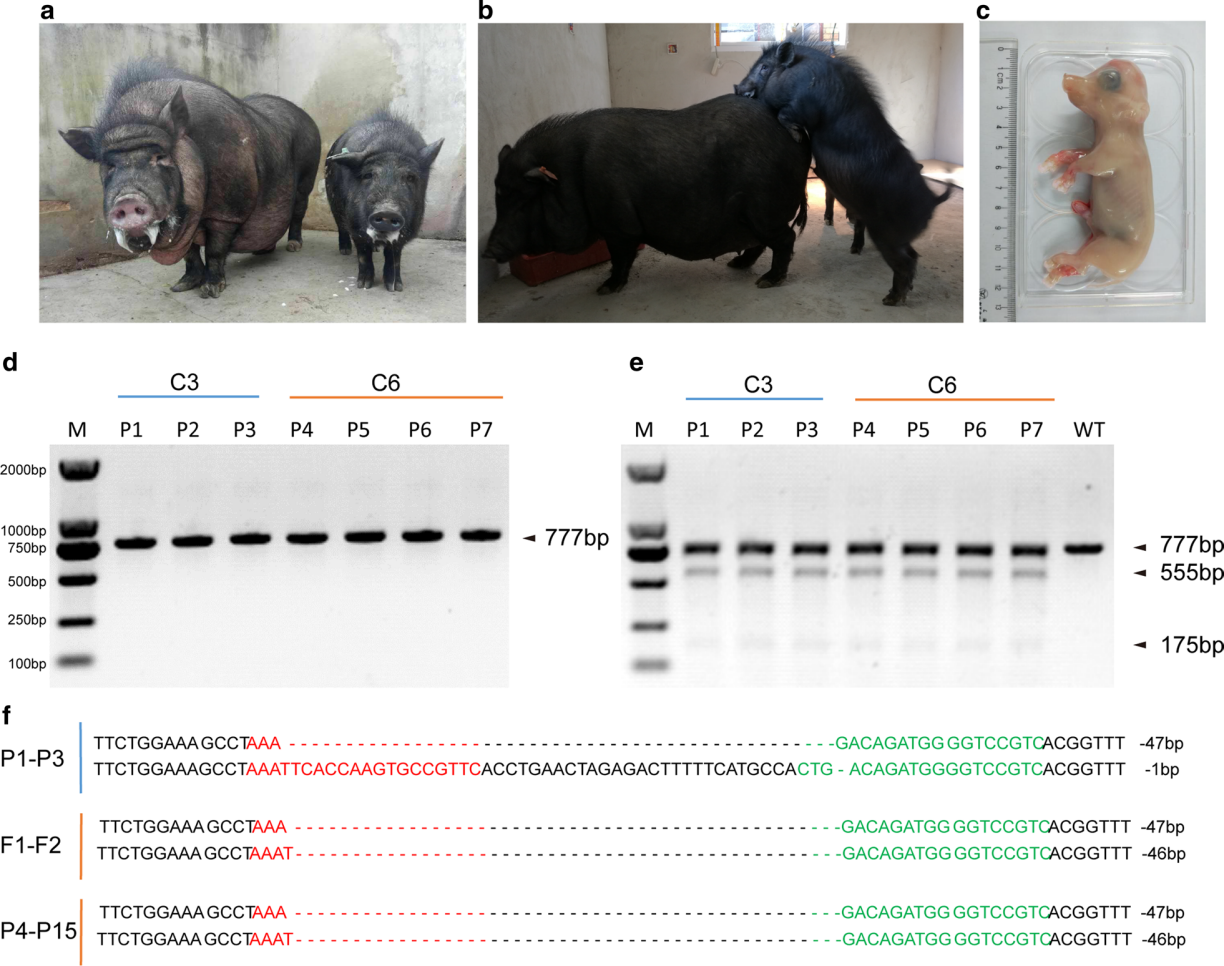
Detection of dual-sgRNAs/Cas9-mediated *GHR* modification in piglets. **a**, **b** Picture of GHRKO pigs showing their small stature and normal fertility. **c** Picture of the live GHRKO fetus that was used to establish cell lines for recloning. **d** PCR products harboring the targeted region of GHR amplified from selected piglets. **e** Detection of dual-RNAs/Cas9-mediated on-target GHR cleavage by the T7ENI cleavage assay in piglets. P1–P3 piglets were cloned from the C3 single-cell colony; P4–P7 piglets were cloned from the C6 single-cell colony. (M, DNA maker DL2000; C, colony; WT, wild-type PCR product digested by T7ENI). **f** Modified GHR sequences detected in piglets and fetuses. F1 and F2 indicate the two fetuses cloned from the C6 cell colony. P4–P15 were cloned piglets from C6 cell colony

According to the growth status, single-cell colony C3 (GHR-47/-1) and single-cell colony C6 (GHR-47/-46) were selected as donor cells for SCNT. The reconstructed embryos were transferred into 6 recipient gilts, with 4 recipients becoming pregnant. Three live piglets derived from C3 cell clones were delivered by one recipient (Table 1). Four live piglets derived from C6 cell clones were delivered by two recipients (Table 1, Fig. 3a, b). We then demonstrated that the 7 cloned piglets described above were all GHRKO pigs by the T7ENI cleavage assay and Sanger sequencing analysis (Fig. 3d–f). The last pregnant recipient returned to estrous 63 days after embryo transfer according to her behavior characteristics. Unintentionally, we obtained two fetuses (one is alive, Fig. 3c) from this recipient gilt when it was used to perform the next embryo transfer as the recipient gilt. The genotypes of these two fetuses were identified by Sanger sequencing, indicating that they were derived from C6 cell clones (Fig. 3f). We then isolated fibroblasts from the live fetus for recloning. The recloned embryos were transferred into 9 recipients, of which 6 became pregnant, and 4 recipients delivered 8 piglets (4 piglets are living). Interestingly, recipient #15 also returned to estrous 49 days after embryo transfer. We determined one live fetus in utero by surgery, and the fetus was successfully delivered at 117 days (Table 1). Three piglets (P1–P3) and 12 piglets (P4–P15) were derived from C3 and C6 donor cells, respectively, which was confirmed by Sanger sequencing analysis (Fig. 3f). Phenotypically, the GHRKO pigs exhibited smaller statures and normal reproductive capacity (Fig. 3a, b).

**Table 1:** Development of reconstructed GHRKO cloned embryos after transfer to recipient gilts

Recipients	Donor cell	No. of transferred embryos	Pregnancy (%)	Days of pregnancy	No. of offspring (alive)
1	C3	236	+	121	3 (3)
2	C6	200	+	118	1
3		200	+	118	3 (3)
4		210	−		
5		210	−		
6^a^		350	+	63	2 (1), fetuses
7	Recloning with alive fetus derived from C6	220	−		
8		215	+	119	1
9		215	−		
10		210	+		
11		220	+	118	2 (1)
12		210	+		
13		157	+	121	4 (2)
14		210	−		
15^a^		210	+	117	1 (1)
Total		218.2 ± 40.1	10 (66.7%)		15 (10)

^a^The pregnant recipients #6 and #15 determined by b-scan returned to estrous 63 days and 49 days after embryo transfer, respectively. The living fetus from recipient #6 was used for the isolation of fibroblast for recloning. The fetus detected by surgery in recipient #15 developed until birth 117 days after embryo transfer

### Evaluation of GHR expression in pigs

**Fig. 4:**
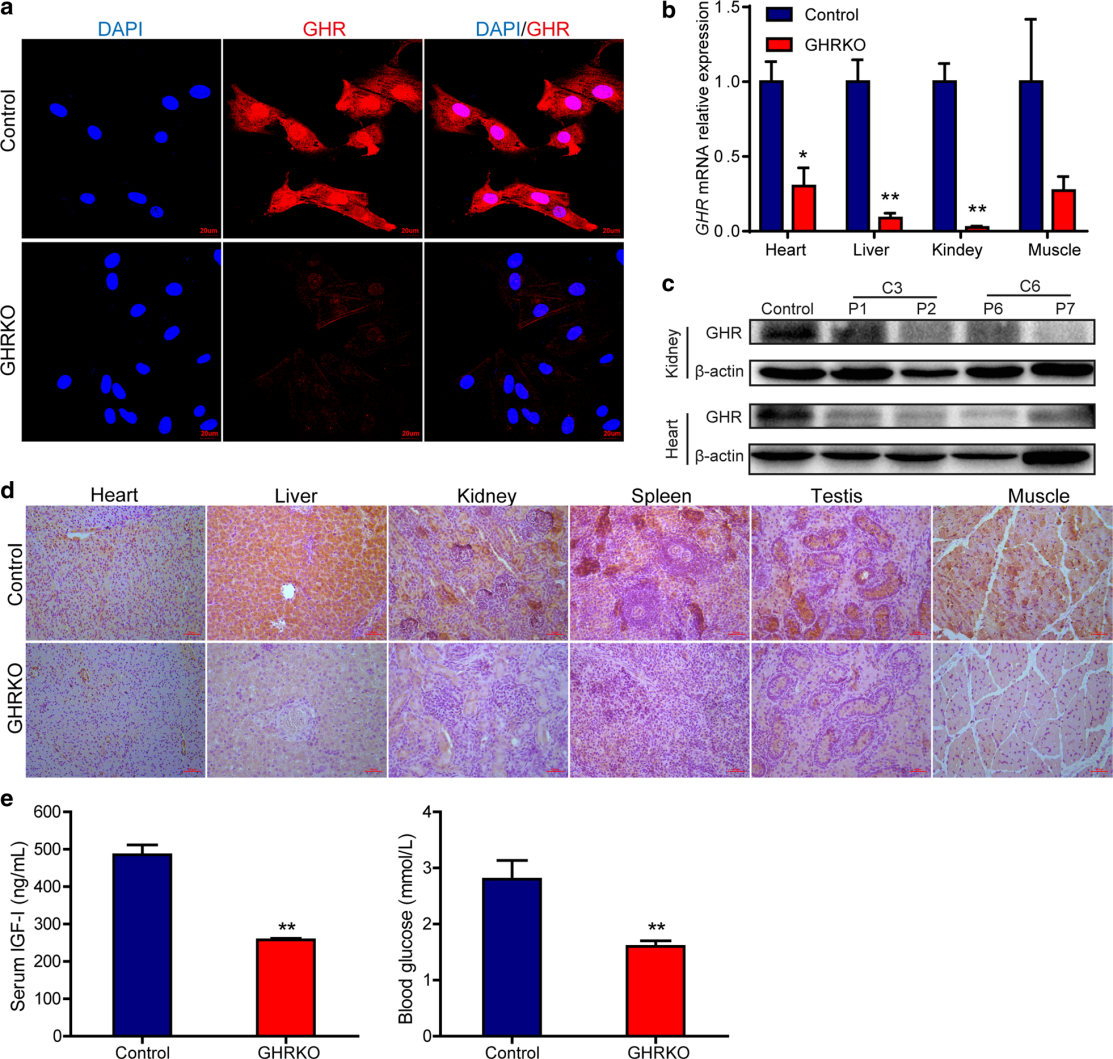
Evaluation of GHR expression in GHRKO pigs. **a** Immunofluorescence analysis of GHR in fibroblasts isolated from GHRKO piglets. Fibroblasts were stained with anti-GHR secondary antibodies (red). DAPI (blue) staining indicates the nucleus. The data are representative of at least three independent experiments. **b** Relative expression of *GHR* mRNA in tissues. Relative GHR expression was detected in heart, liver, kidney and muscle tissues. GAPDH served as the internal control. The data were derived from four GHRKO pigs and three wildtype pigs; the bars represent the mean ± SEM; *P < 0.05 and **P < 0.01. **c** Immunoblotting analysis of porcine GHR in heart and kidney tissues. Protein extracted from wild-type pig was used as the control. β-Actin was used as the internal control. **d** Immunochemical analysis of various tissues from GHRKO pigs. Heart, liver, kidney, spleen, testis and muscle tissues from GHRKO pigs were GHR-deficient. Wild-type pigs were used as the control. **e** Compared to those in control pigs, serum IGF-I and blood glucose levels were significantly decreased in GHRKO pigs, which was consistent with that observed in Laron patients; **P < 0.01

We further evaluated the expression of *GHR* at the transcription and translation levels, as determining whether *GHR* mutations result in protein dysfunction and induce Laron syndrome phenotypes is important. We first analyzed *GHR* protein expression in fibroblasts isolated from GHRKO piglets (Fig. 4a), revealing that they were GHR-deficient. We further investigated the *GHR* mRNA expression levels in heart, liver, kidney and muscle tissues from GHRKO pigs and compared them to those of wild-type control pigs, demonstrating that the *GHR* mRNA relative expression levels in GHRKO pigs were significantly lower than those in the control in the three tissues analyzed (*P* < 0.05, Fig. 4b). Similarly, investigation of *GHR* protein expression via western blotting and antibody immunostaining indicated that the *GHR* protein in GHRKO piglets was defective compared to that of the controls (Fig. 4c, d), as a loss of *GHR* protein expression was observed in the different tissues. Furthermore, the serum IGF-I levels were significantly lower in GHRKO pigs than those in wild-type control pigs (*P* < 0.01). Significantly low levels of glucose were also observed in GHRKO pigs (Fig. 4e), which was consistent with hypoglycemia observed in Laron patients. Overall, these results suggest that the GHR gene was dysfunctional in these GHRKO piglets.

### GHRKO pigs exhibited growth retardation and abnormal biochemical features

**Fig. 5:**
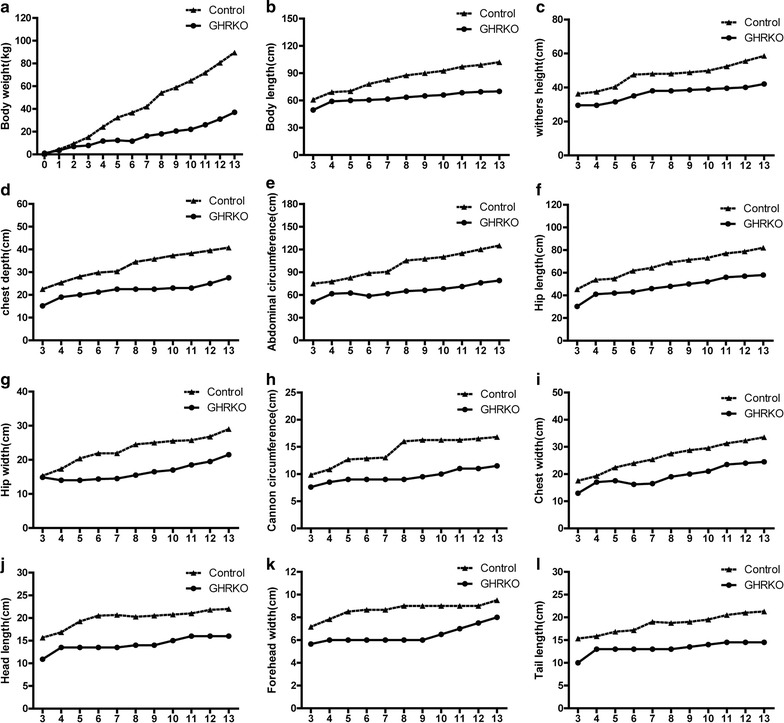
Growth retardation in GHRKO pigs. **a**–**l** Parameters of pig growth, including body weight, body length, withers height, chest depth, abdominal circumference, hip length, hip width, cannon circumference, chest width, head length, forehead width and tail length. The abscissa number indicates the age of the pig in months

To accurately assay growth characteristics induced by biallelic *GHR* mutation in GHRKO pigs, we carefully documented growth parameters, including body weight, body length, withers height, chest depth, abdominal circumference, hip length, hip width, cannon circumference, chest width, head length, forehead width and tail length. The GHRKO pigs had slow growth rates and small body sizes. From birth to 13 months old, the average body weights of wild-type pigs varied from 0.6 to 89.5 kg, while those of GHRKO pigs varied only from 0.9 to 37.0 kg (Fig. 5a). The body size variability of GHRKO pigs was also smaller than that of wild-type pigs (Fig. 5b–i).

### Germline transmission

**Fig. 6:**
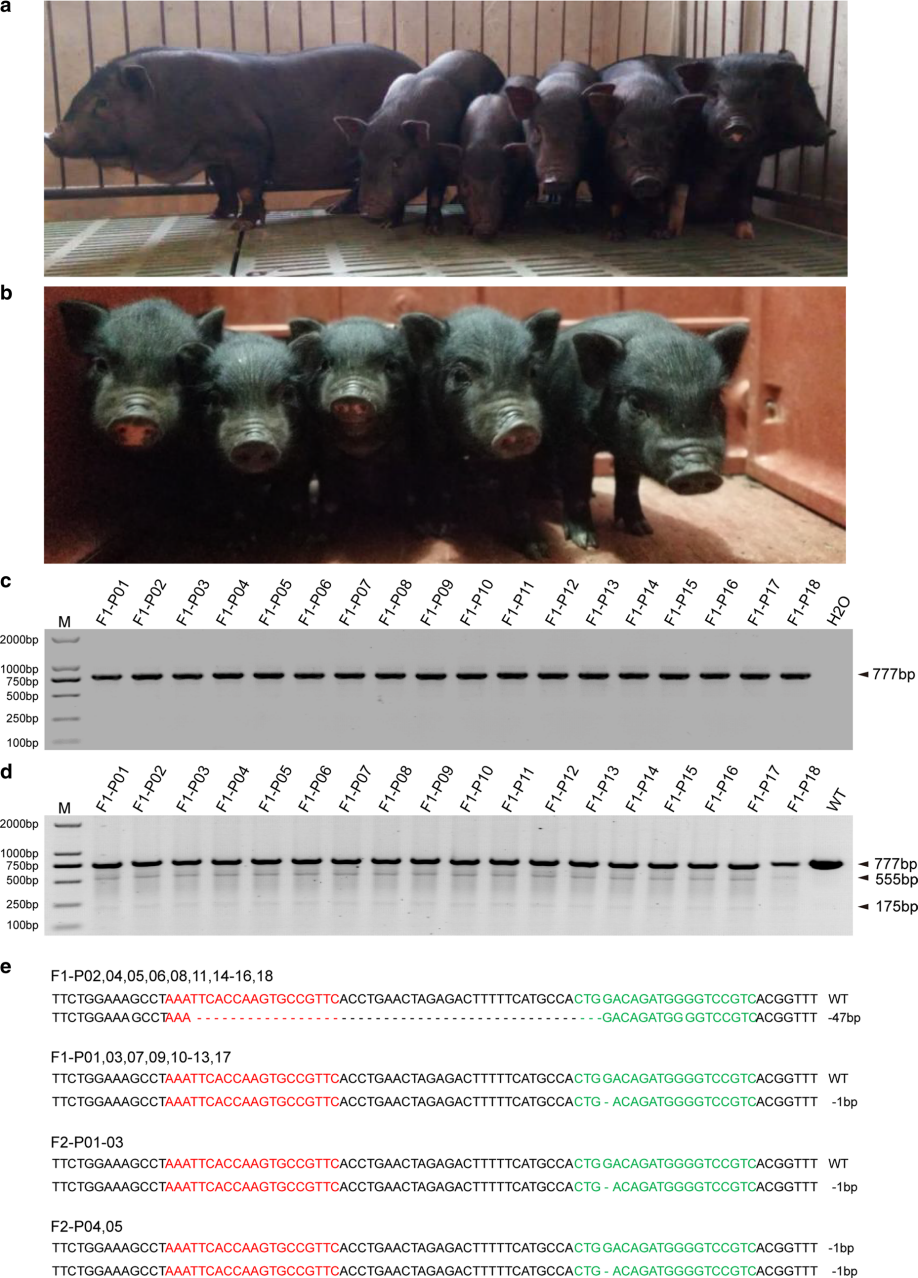
Germline transmission of *GHR*-modified alleles. **a** Picture of partial GHRKO F1 pigs; the leftmost pig is wild-type. **b** Picture of GHRKO F2 pigs. **c** PCR products harboring the targeted *GHR* region amplified from GHRKO F1 pigs. **d** Detection of the *GHR* genotype by the T7ENI cleavage assay in GHRKO F1 pigs (M, DNA maker DL2000; WT, PCR product amplified from wild-type pig digested by T7ENI). **e** Sequences of modified *GHR* detected in GHRKO F1 and F2 pigs

A male GHRKO pig (P2) was mated with wild-type female pigs, and 18 piglets were obtained (Fig. 6a). We detected the *GHR* genotype of F1 offspring by the T7ENI cleavage assay, TA cloning and Sanger sequencing (Fig. 6 c–e). Monoallelic *GHR* modifications were found in 18 GHRKO F1 piglets, and modified *GHR* sequences were identified, showing a 47-bp deletion in 10 piglets and a 1-bp deletion in 8 piglets (Fig. 6e). We further obtained a total of five F2 generation piglets delivered by self-cross of F1 generation (Fig. 6b). Two F2 piglets were identified as homozygous mutation at *GHR* loci harboring 1 bp deletion according to Sanger sequencing. Another three piglets were heterozygote at the targeting site (Fig. 6e). Above results showed that the modified *GHR* allele stably pass to the next generation via germline transmission. Thus, the modified *GHR* allele was able pass to the next generation via germline transmission.

## Discussion

Pigs are a suitable biomedical model to fill gaps between mouse models and human patients, with a potential for increased utilization in diverse lines of research [29]. Regarding Laron syndrome, many similarities in bone growth dynamics [30] and glucose and lipid metabolism exist [10]. Importantly, pigs also possess many similarities to humans at the GH-IGF-I axis [31]. Therefore, the Laron pig model enables the elucidation of many aspects of long-standing IGF-I deprivation in a variety of organs and tissues. Taken together, the Laron pig is an improved model for studying the effects of GHR defects on growth and metabolism observed in Laron syndrome and for assessing the efficacy of Laron syndrome treatments.

The CRISPR/Cas9 system has been developed into a highly efficient and flexible genome editing tool with wide-ranging applications. In recent publications, dual-sgRNAs/Cas9 systems were designed to generate large gene deletions, including long non-coding RNAs and gene regulatory regions [32, 33]. Furthermore, such gene editing systems showed enhanced gene targeting efficiency due to the use of multiple sgRNAs [34]. Here, we designed a dual-sgRNAs/Cas9 system to target exon 3 of the *GHR* gene, which significantly improved the Cas9-mediated genome targeting, as a biallelic modification efficiency of up to 95% was achieved. Moreover, the major type of mutation in the *GHR* gene was a 47-bp deletion, consistent with the prospective target mutation, suggesting that the dual-sgRNAs/Cas9 system can generate precise gene fragment deletions.

Loss of function in *GHR* genes, predominantly owing to mutations in their extracellular domains, results in Laron syndrome [35]. In the present study, exon 3 of GHR was disrupted, and the mRNA and protein expression levels of *GHR* were dramatically reduced in GHRKO pigs. The result suggests that such deletions in exon 3 of the GHR gene influence the entire GHR gene instead of only its extracellular domain. The GHRKO pigs showed severe postnatal growth retardation according to their body weights and sizes. Furthermore, the GHRKO pigs exhibited significantly reduced serum levels of IGF-I and blood glucose, which was consistent with the pathological characteristics of Laron patients. However, the GHRKO pigs showed no discernible growth phenotype at birth when compared with wild-type pigs, which was also observed in recent publications [4, 36]. It is generally accepted that GH has no direct regulatory roles in fetal growth, while IGF-I exerts its fundamental effects on prenatal growth both via endocrine and paracrine signaling [37]. In addition, the placenta is a metabolically active tissue that can secrete hormones into maternal and fetal circulation. Handwerger and Freemark [38] believe that a GH variant expressed by the placenta (hGH-V) rather than the pituitary fetus stimulates IGF production and modulates intermediary metabolism, resulting in fetal growth. The accumulation of hGH-V in the placenta due to lacking GHR might affect maternal reproduction hormones [39], thus influencing the return to estrous in the gestational gilts observed in this study. Laron patients are known to have delayed puberty, but our GHRKO pigs showed early puberty. Thus, we detected the testosterone concentration in GHRKO and WT pigs, the result showed the testosterone concentration of a GHRKO pig was over twofold compared with that of WT pigs (data not shown). However, whether the early puberty in GHRKO pigs is a common feature, which requires further investigation. Furthermore, the fertility of the GHRKO pigs was normal, and the modified *GHR* alleles could pass to the next generation via germline transmission. This is important for expanding the population of *GHR*-modified individuals.

## Conclusions

In conclusion, we achieved precisely targeted DNA deletion with high efficiency using a dual-sgRNAs/Cas9 system in pigs, suggesting that dual-sgRNAs/Cas9 is a reliable system for DNA deletion. The dual-sgRNAs/Cas9 genome editing system combined with SCNT represents a highly efficient approach for generating valid pig models of human diseases. Here, GHRKO pigs recapitulated human Laron syndrome caused by mutations in *GHR*. Therefore, GHRKO pigs will be a very useful disease model for areas of long-term development for which human data are still limited or lacking and for new therapeutic developments.

## Additional file

**Additional file 1: Table S1.** Oligonucleotides for generating sgRNA expression vectors. **Table S2.** Primers for genotyping and amplifying targeted *GHR* fragments. **Table S3.** Sequences of primers for the q-PCR amplification of *GHR.* **Table S4.** Summary of sgRNAs/Cas9-mediated *GHR* genotypes modified in single-cell colonies.

## Abbreviations

GHR: growth hormone receptor
IGF-I: insulin-like growth factor I
GHRKO: GHR knockout
PFFs: pig fetal fibroblasts
SCNT: somatic cell nuclear transfer
T7ENI: T7 endonuclease I
q-PCR: quantitative polymerase chain reaction
WB: western blotting
IHC: immunohistochemical
CRISPR/Cas9: clustered regularly interspaced short palindromic repeats/CRISPR associated 9
DMEM: Dulbecco’s modified Eagle’s medium
FBS: fetal bovine serum
IVM: *in vitro* maturation
COCs: cumulus-oocyte complexes
EDTA: ethylenediaminetetraacetate
DAPI: 4′,6-diamidino-2-phenylindole
PBS: phosphate buffered solution
RIPA: radio immunoprecipitation assay
SDS-PAGE: sodium dodecyl sulfate polyacrylamide gel electrophoresis
PVDF: polyvinylidene difluoride
ECL: chemiluminescence
ELISA: enzyme-linked immunosorbent assay
PZM-3: porcine zygote medium-3
SD: standard deviation
SPSS: Statistical Product and Service Solutions
HRP: horseradish peroxidase
TMB: 3,3′,5,5′-tetramethylbenzidine
