# Supplementary material for: Generation of GHR-modified pigs as Laron syndrome models via a dual-sgRNAs/Cas9 system and somatic cell nuclear transfer
Source: J Transl Med. 2018 Feb 27;16:41. doi: 10.1186/s12967-018-1409-7 (PMC5828148; doi:10.1186/s12967-018-1409-7)
Supplement: Supplementary file 1 — Additional file 1: Table S1. Oligonucleotides for generating sgRNA expression vectors. Table S2. Primers for genotyping and amplifying targeted GHR fragments. Table S3. Sequences of primers for the q-PCR amplification of GHR. Table S4. Summary of sgRNAs/Cas9-mediated GHR genotypes modified in single-cell colonies. [file 12967_2018_1409_MOESM1_ESM.docx]

**Table S1** Oligonucleotides for generating sgRNA expression vector.

| **Oligonucleotides** | **Sequence** |
| --- | --- |
| sgRNA1 top strand | 5’-GAACGGCACTTGGTGAATTT-3’ |
| sgRNA1 bottom strand | 5’-AAATTCACCAAGTGCCGTTC-3’ |
| sgRNA2 top strand | 5’-GACGGACCCCATCTGTCCAG-3’ |
| sgRNA2 bottom strand | 5’-CTGGACAGATGGGGTCCGTC-3’ |
| sgRNAc top strand | 5’-GGCAAGCCGTTGCTGATTCG-3’ |
| sgRNAc bottom strand | 5’-CGAATCAGCAACGGCTTGCC-3’ |

**Table S2** Primers for genotyping and amplifying targeted *GHR* fragment.

| **Name** | **Sequence** | **Amplicon** |
| --- | --- | --- |
| pGHR For | 5’- CGACAGAGGAATGATTGACAAGAAC-3’ | 777 |
| pGHR Rev | 5’- CACATTCACCTACTTTCCCACATTC-3’ |  |

**Table S3** Sequences of primers for Q-PCR amplification of the *GHR.*

| **Name** | **Sequence** |
| --- | --- |
| pGHR For | 5’-ACCTCCCAATGCAGATGTTCAG-3’ |
| pGHR Rev | 5’-AATGGGCTCATCTGAGGAAGTG-3’ |

**Table S4** Summary of the sgRNAs/Cas9-mediated modified *GHR* genotype in single cell clones.

| **Cell No.** | **Sequence** | **Deletion (-)**  **NO. of base pairs** | **Positive sequencing**  **count** |
| --- | --- | --- | --- |
| WT | 5’ GCCTAAATTCACCAAGTGCCGTTCACCTGAACTAGAGACTTTTTCATGCCACTGGACAGATGG 3’ |  |  |
| C1 | 5’ GCCTAAA - - - - - - - - - - - - - - - - - - - - - - - - - - - - - - - - - - - - - - - - - - - - - - - - - - - - - - - -- - - GACAGATGG 3’  5’ GCCTAAAT - - - - - - - - - - - - - - - - - - - - - - - - - - - - - - - - - - - - - - - - - - - - - - - - - - - - -- - - - - -GACAGATGG 3’ | - 47  - 46 | 6  4 |
| C2 | 5’ GCCTAAA - - - - - - - - - - - - - - - - - - - - - - - - - - - - - - - - - - - - - - - - - - - - - - - - - - - - - - - - - - - GACAGATGG 3’  5’ GCCTAAAT - - - - - - - - - - - - - - - - - - - - - - - - - - - - - - - - - - - - - - - - -- - - - - - - - - - - - - - - - - -GACAGATGG 3’ | - 47  - 46 | 6  8 |
| C3 | 5’ GCCTAAA - - - - - - - - - - - - - - - - - - - - - - - - - - - - - - - - - - - - - - - - - - - - - - - - - - - - - - --- - - - GACAGATGG 3’  5’ GCCTAAATTCACCAAGTGCCGTTCACCTGAACTAGAGACTTTTTCATGCCACT - GACAGATGG 3’ | - 47  - 1 | 4  6 |
| C4 | 5’ GCCTAAA - - - - - - - - - - - - - - - - - - - - - - - - - - - - - - - - - - - - - - - - - - - - - - - - - - - - - - - - - - - GACAGATGG 3’  5’ GCCTAAATTCACCAAGTGCCGTTCACCTGAACTAGAGACTTTTTCATG - - - - - - - - - - - - - - - - - -3’ | - 47  - 18 | 2  3 |
| C5 | 5’ GCCTAAA - - - - - - - - - - - - - - - - - - - - - - - - - - - - - - - - - - - - - - - - -- - - - - - - - - - - - - - - - - - - GACAGATGG 3’  5’ GCCTA - - - - - - - - - - - - - - - - - - - - - - - - - - - - - -- - - - - - - - - - - - - - - - - - - - - - - - - - - - - - - GACAGATGG 3’ | - 47  - 49 | 2  1 |
| C6 | 5’ GCCTAAA - - - - - - - - - - - - - - - - - - - - - - - - - - - - - - - - - - - - - - - - - - - - - - - - - - - - - - - - - - - GACAGATGG 3’  5’ GCCTAAAT - - - - - - - - - - - - - - - - - - - - - - - - - - - - - - - - - - - - - - - - - - - - - - - - - -- - - - - - - - -GACAGATGG 3’ | - 47  - 46 | 10  8 |
| C7 | 5’ GCCTAAA - - - - - - - - - - - - - - - - - - - - - - - - - - - - - - - - - - - - - - - - - - - - - - - - - - - - - - - - - - - GACAGATGG 3’  5’ GCCTAAAT - - - - - - - - - - - - - - - - - - - - - - - - - - - - - - - - - - - - - - - - - - - - - - - - - -- - - - - - - - -GACAGATGG 3’ | - 47  - 46 | 4  2 |
| C8 | 5’ GCCTAAA - - - - - - - - - - - - - - - - - - - - - - - - - - - - - - - - - - - - - - - - - - - - - - - - - - - - - - - - - - - GACAGATGG 3’  5’ GCCTAAAT - - - - - - - - - - - - - - - - - - - - - - - - - - - - - - - - - - - - - - - - - - - - - - - - - -- - - - - - - - -GACAGATGG 3’ | - 47  - 46 | 6  4 |
| C9 | 5’ GCCTAAA - - - - - - - - - - - - - - - - - - - - - - - - - - - - - - - - - - - - - - - - - - - - - - - - - - - - - - - - - - - GACAGATGG 3’  5’ GCCTAAAT - - - - - - - - - - - - - - - - - - - - - - - - - - - - - - - - - - - - - - - - - - - - - - - - - -- - - - - - - - -GACAGATGG 3’ | - 47  - 46 | 7  7 |
| C10 | 5’ GCCTAAA - - - - - - - - - - - - - - - - - - - - - - - - - - - - - - - - - - - - - - - - - - - - - - - - - - - - - - - - - - - GACAGATGG 3’  5’ GCCTAAAT - - - - - - - - - - - - - - - - - - - - - - - - - - - - - - - - - - - - - - - - - - - - - - - - - -- - - - - - - - -GACAGATGG 3’ | - 47  - 46 | 6  3 |
| C11 | 5’ GCCTAAA - - - - - - - - - - - - - - - - - - - - - - - - - - - - - - - - - - - - - - - - - - - - - - - - - - - - - - - - - - - GACAGATGG 3’  5’ GCCTAAAT - - - - - - - - - - - - - - - - - - - - - - - - - - - - - - - - - - - - - - - - - - - - - - - - - -- - - - - - - - -GACAGATGG 3’ | - 47  - 46 | 5  3 |
| C12 | 5’ GCCTAAAT - - - - - - - - - - - - - - - - - - - - - - - - - - - - - - - - - - - - - - - - - - - - - - - - - -- - - - - - - - -GACAGATGG 3’  5’ GCCTA - - - - - - - - - - - - - - - - - - - - - - - - - - - -- - - - - - - - - - - - - - - - - - - - - - - - - - - - - - - - - - GACAGATGG 3’ | - 46  - 49 | 3  4 |
| C13 | 5’ GCCTAAA - - - - - - - - - - - - - - - - - - - - - - - - - - - - - - - - - - - - - - - - - - - - - - - - - - - - - - - - - - - GACAGATGG 3’  5’ GCCTAAAT - - - - - - - - - - - - - - - - - - - - - - - - - - - - - - - - - - - - - - - - - - - - - - - - - -- - - - - - - - -GACAGATGG 3’ | - 47  - 46 | 7  3 |
| C14 | 5’ GCCTAAA - - - - - - - - - - - - - - - - - - - - - - - - - - - - - - - - - - - - - - - - - - - - - - - - - - - - - - - - - - - GACAGATGG 3’  5’ GCCTAAAT - - - - - - - - - - - - - - - - - - - - - - - - - - - - - - - - - - - - - - - - - - - - - - - - - -- - - - - - - - -GACAGATGG 3’ | - 47  - 46 | 8  5 |
| C15 | 5’ GCCTAAA - - - - - - - - - - - - - - - - - - - - - - - - - - - - - - - - - - - - - - - - - - - - - - - - - - - - - - - - - - - GACAGATGG 3’  5’ GCCTAAAT - - - - - - - - - - - - - - - - - - - - - - - - - - - - - - - - - - - - - - - - - - - - - - - - - -- - - - - - - - -GACAGATGG 3’ | - 47  - 46 | 9  2 |
| C16 | 5’ GCCTAAA - - - - - - - - - - - - - - - - - - - - - - - - - - - - - - - - - - - - - - - - - - - - - - - - - - - - - - - - - - - GACAGATGG 3’  5’ GCCTAAAT - - - - - - - - - - - - - - - - - - - - - - - - - - - - - - - - - - - - - - - - - - - - - - - - - -- - - - - - - - -GACAGATGG 3’ | - 47  - 46 | 6  2 |
| C17 | 5’ GCCTAAA - - - - - - - - - - - - - - - - - - - - - - - - - - - - - - - - - - - - - - - - - - - - - - - - - - - - - - - - - - - GACAGATGG 3’  5’ GCCTAAAT - - - - - - - - - - - - - - - - - - - - - - - - - - - - - - - - - - - - - - - - - - - - - - - - - -- - - - - - - - -GACAGATGG 3’ | - 47  - 46 | 7  4 |
| C18 | 5’ GCCTAAA - - - - - - - - - - - - - - - - - - - - - - - - - - - - - - - - - - - - - - - - - - - - - - - - - - - - - - - - - - - GACAGATGG 3’  5’ GCCTAAAT - - - - - - - - - - - - - - - - - - - - - - - - - - - - - - - - - - - - - - - - - - - - - - - - - -- - - - - - - - -GACAGATGG 3’ | - 47  - 46 | 6  2 |
| C19 | 5’ GCCTAAATTCACCAAGTGCCGTTCACCTGAACTAGAGACTTTTTCATGCCACTGGACAGATGG 3’ | 0 | 8 |
| C20 | 5’ GCCTAAA - - - - - - - - - - - - - - - - - - - - - - - - - - - - - - - - - - - - - - - - - - - - - - - - - - - - - - - - - - - GACAGATGG 3’  5’ GCCTAAAT - - - - - - - - - - - - - - - - - - - - - - - - - - - - - - - - - - - - - - - - - - - - - - - - - -- - - - - - - - -GACAGATGG 3’ | - 47  - 46 | 5  2 |
